# 

**Published:** 1896-12

**Authors:** 


					if==
DECEMBER, 1896.
Vol. XIV. No. 54.
THE
BRISTOL
Lu /
MEDICO-CHIRURGICAL
tfSOiV/iM rrp
B (Sluartcrlg Journal of tbe /Ifoefctcal Sciences for tbe West
of BitglanD an?> Soutb Wales
PUBLISHED UNDER THE AUSPICES OF
THE BRISTOL MEDICO-CHIRURGICAL SOCIETY.
"Scire est nescire, nisi 15 me
Scire alius sciret."
Editor: R. SHINGLETON SMITH, M.D., B.Sc.
WITH WHOM ARE ASSOCIATED
J. MICHELL CLARKE, M.A., M.D.
F. R. CROSS, M.B. and W. H. HARSANT.
Assistant-Editor: L. M. GRIFFITHS.
Editorial Secretary: B. M. H. ROGERS, B.A., M.D., B.Ch.
BRISTOL: J. W. ARROWSMITH ;
London : J. & A. Churchill, 7 Great Marlborough Street.
Price One Shilling and Sixpence.

				

